# Insights into the Cardioprotective Effects of Pyridoxine Treatment in Diabetic Rats: A Study on Cardiac Oxidative Stress, Cardiometabolic Status, and Cardiovascular Biomarkers

**DOI:** 10.3390/diagnostics14141507

**Published:** 2024-07-12

**Authors:** Slavica Mutavdzin Krneta, Kristina Gopcevic, Sanja Stankovic, Jovana Jakovljevic Uzelac, Dušan Todorovic, Milica Labudovic Borovic, Jelena Rakocevic, Dragan Djuric

**Affiliations:** 1 Institute of Medical Physiology “Richard Burian”, Faculty of Medicine, University of Belgrade, 11000 Belgrade, Serbia; jovanavjakovljevic@gmail.com (J.J.U.); t.dusan@hotmail.com (D.T.); dr_djuric@yahoo.com (D.D.); 2 Institute of Chemistry in Medicine “Prof. Dr. Petar Matavulj”, Faculty of Medicine, University of Belgrade, 11000 Belgrade, Serbia; kristinagopcevic@yahoo.com; 3 Centre for Medical Biochemistry, University Clinical Centre of Serbia, 11000 Belgrade, Serbia; sanjast2013@gmail.com; 4 Department of Biochemistry, Faculty of Medical Sciences, University of Kragujevac, 34000 Kragujevac, Serbia; 5 Institute of Histology and Embryology “Aleksandar Dj. Kostic”, Faculty of Medicine, University of Belgrade, 11000 Belgrade, Serbia; sborovic2001@yahoo.com (M.L.B.); jelena.rakocevic@med.bg.ac.rs (J.R.)

**Keywords:** cardiac oxidative stress, diabetes mellitus, pyridoxine, rat, streptozotocin

## Abstract

The aims of this study were to examine the effects of pyridoxine administration on the activities of cardiac antioxidant stress enzymes superoxide dismutase (SOD) and catalase (CAT) and enzyme indicators of cardiometabolic status, lactate and malate dehydrogenase (LDH, MDH), as well as LDH and MDH isoforms’ distribution in the cardiac tissue of healthy and diabetic Wistar male rats. Experimental animals were divided into five groups: C1—control (0.9% sodium chloride—NaCl—1 mL/kg, intraperitoneally (i.p.), 1 day); C2—second control (0.9% NaCl 1 mL/kg, i.p., 28 days); DM—diabetes mellitus (streptozotocin 100 mg/kg in 0.9% NaCl, i.p., 1 day); P—pyridoxine (7 mg/kg, i.p., 28 days); and DM + P—diabetes mellitus and pyridoxine (streptozotocin 100 mg/kg, i.p., 1 day and pyridoxine 7 mg/kg, i.p., 28 days). Pyridoxine treatment reduced CAT and MDH activity in diabetic rats. In diabetic rats, the administration of pyridoxine increased LDH1 and decreased LDH4 isoform activities, as well as decreased peroxisomal MDH and increased mitochondrial MDH activities. Our findings highlight the positive effects of pyridoxine administration on the complex interplay between oxidative stress, antioxidant enzymes, and metabolic changes in diabetic cardiomyopathy.

## 1. Introduction

Diabetes mellitus (DM) ranks among the most prevalent metabolic disorders. This chronic metabolic condition is marked by persistently high blood glucose levels, which, over time, can cause damage to many organs and systems, such as the cardiovascular system, nervous system, eyes, and kidneys [1]. Diabetic cardiomyopathy manifests as myocardial dysfunction in individuals with diabetes mellitus, irrespective of concurrent coronary artery disease, valvular pathology, dyslipidemia, or systemic hypertension. It is characterized by cardiomyocyte hypertrophy, myocardial fibrosis, cardiomyocyte apoptosis, cardiac remodeling, and capillary basement membrane thickening. Typically, it initiates with a latent phase of diastolic dysfunction, which later can progress to systolic dysfunction and heart failure with decreased ejection fraction [2,3,4].

Lately, it has been shown that higher oxidative stress and alterations in antioxidant defense mechanisms are important in the development of DM and its complications [5,6]. Oxidative stress is a pivotal factor in the pathogenesis of atherosclerosis, endothelial dysfunction, and the progression of cardiovascular diseases [7]. Oxidative damage is considered to be a major mechanism of endothelial dysfunction in DM type 1 [8], so the administration of substances that will decrease oxidative stress is recommended.

In our previous studies, we demonstrated that the induction of DM in rats leads to numerous cardiometabolic changes [9,10]. Among these changes, it is important to note alterations in the activities of antioxidant enzymes such as superoxide dismutase (SOD) and catalase (CAT) and indicators of tissue metabolic status enzymes (lactate dehydrogenase—LDH—and malate dehydrogenase—MDH) [9]. Following treatment with folic acid, positive effects on the altered parameters were observed. Additionally, a strong antidiabetic effect of folic acid was noted, considering the significant reduction in glucose levels in rats with DM after folic acid administration [9,10].

In this study, our objective was to assess the impact of administering vitamin B6 (pyridoxine) on both healthy rats and those with experimentally induced diabetes mellitus (DM). This investigation was prompted by existing evidence suggesting that vitamin B6 possesses antioxidant properties, despite its non-classification as a conventional antioxidant agent [11]. Pyridoxine has an important role in complete metabolism [12]. It has been demonstrated that vitamin B6 inhibits lipid peroxidation and has an important role in antioxidant defense [13]. There are indications that a deficiency in vitamin B6 is associated with the development of atherosclerosis [12].

The metabolic changes that occur in DM can be examined by the assessment of LDH and MDH isoform activities. Five LDH isoforms may be detected (LDH1-LDH5) that differ in enzymatic activity and composition. Isoforms LDH1 and LDH2 primarily catalyze the oxidation of lactate to pyruvate during aerobic conditions, while LDH5 and LDH4 promote the reduction of pyruvate to lactate, and therefore, they are essential for glycolysis at low oxygen levels [14,15]. MDH catalyzes the oxidation of malate into oxaloacetate and the reduction of oxaloacetate into malate in the Krebs cycle [16], so it is directly implicated in the metabolism of glucose. This enzyme has two main isoforms, mitochondrial (mMDH) and cytosolic (cMDH); however, the third peroxisomal (pMDH) isoform can also be detected [16,17]. The presence of MDH in peroxisomes may indicate a malate shuttle mechanism across the peroxisomal membrane in vertebrate cells, analogous to the mitochondrial aspartate–malate shuttle, facilitating NADH generation in a manner similar to mitochondrial MDH [17].

Despite the previously published results, the effect of pyridoxine on the antioxidant defense and metabolic changes in the cardiac tissue of healthy rats and rats with DM type I is not completely clear. Therefore, the objective of this study was to investigate if pyridoxine administration affects DM by influencing the cardiometabolic biomarkers, antioxidant enzymes, total LDH and MDH activities, and their isoform activities in the cardiac tissue and the histomorphometric and immunohistochemical parameters of cardiac tissue.

## 2. Materials and Methods

### 2.1. Experimental Animals

This research was conducted using Wistar albino rats (male, body mass around 160 g in the beginning). They were housed in transparent Plexiglas cages with a floor covered in woodchips. Conventional rat feed and water were available ad libitum. The ambient conditions were a temperature of 21 ± 2 °C, a relative humidity of 55 ± 5%, and a 12/12 h light–dark cycle with the light period beginning at 07:30 a.m. All experimental procedures were performed according to the Guide for the Care and Use of Laboratory Animals [18], European Directive for the Protection of Vertebrate Animals used for Experimental and other Scientific Purposes (86/609/EES), and ethics principles. This research was permitted by the Ethical Council for the Welfare of Experimental Animals, Ministry of Agriculture, Forestry and Water Management, Veterinary Directorate, Republic of Serbia (No: 323-07-01339/2017-05/04).

### 2.2. Experimental Protocol

At the beginning of the experimental period that lasted four weeks, the animals were randomly divided into five groups. The first group (C1, *n* = 8) received one dose of 0.9% NaCl (physiological saline, 1 mL/kg b.w., intraperitoneally—i.p.), and the second group (C2, *n* = 10) received treatment with 0.9% NaCl for 28 successive days (1 mL/kg b.w., i.p.). The C2 group was introduced to test if daily 0.9% NaCl treatment may affect the examined variables. The third group was the DM group (DM, *n* = 8) in which DM was induced by one dose of streptozotocin (100 mg/kg b.w. in 0.9% NaCl, i.p.). The fourth group, the pyridoxine group (P, *n* = 8), received pyridoxine (7 mg/kg b.w. in 0.9% NaCl, i.p.) treatment for 28 successive days. The fifth group was the group of experimental animals with induced DM and pyridoxine treatment (DM + P, *n* = 8). They received streptozotocin (100 mg/kg b.w. in 0.9% NaCl, i.p., in one dose), and on the fourth day after streptozotocin injection, they were treated with pyridoxine (7 mg/kg b.w. in 0.9% NaCl, i.p.) for the next 28 successive days.

The recommended daily intake of vitamin B6 is 6 to 7 mg/kg in rats [19]. In our study, we wanted to examine whether, in addition to vitamin B6 intake by standard food, the supplemental administration of pyridoxine at the daily recommended dose (7 mg/kg) may have positive effects both in healthy rats and in rats with diabetes mellitus.

This study measured body mass and rats’ blood glucose levels weekly throughout the testing period. At the end of the experimental period, animals were euthanized using a rat guillotine. Animals were fasted overnight (12 h) before euthanasia to minimize food-induced changes in blood biochemical parameters. Blood samples were collected by exsanguination for serum or plasma isolation. Vacutainers without anticoagulants were used for serum isolation, while vacutainers with sodium citrate (anticoagulant) were used for plasma isolation. Samples were left at room temperature for 15 min and then centrifuged at 1000× *g* for another 15 min to obtain plasma and serum for analysis. After blood collection, hearts were isolated, rinsed in 0.9% NaCl, and their mass was measured. Half of the hearts from each group were fixed in formalin for subsequent histological analysis, while the other half were homogenized and centrifuged to assess enzyme activity.

### 2.3. Induction of Diabetes Mellitus in Rats

DM was induced by an i.p. injection of streptozotocin (100 mg/kg b.w.), as previously described in our studies [9,10].

### 2.4. Blood, Serum, and Plasma Biochemical Parameters Determination

For weekly glucose level analysis, blood was obtained from the rat tail vein. Before the measurement, the animals were fasted for 8 h. For the measurements, an Accu-Chek analyzer (Roche Diagnostics, Indianapolis, IN, USA) was used. Serum homocysteine was evaluated by an automated electrochemiluminescence immunoassay system, ADVIA Centaur XP system (Siemens Healthcare Diagnostics, New York, NY, USA). The serum glucose level, lipid profile parameters (total cholesterol—TC; cholesterol—HDL-C; triglycerides—TG), and cardiac tissue injury parameter (LDH) were analyzed using a spectrophotometer and commercial kits (Siemens Healthcare Diagnostics Inc., Newark, NJ, USA) on an automatic biochemical analyzer (Dimension Xpand, Siemens, Washington, DC, USA). The low-density lipoprotein cholesterol (LDL-C) serum level was estimated using the Friedewald equation. The concentration of fibrinogen in citrated plasma was measured by the modified Klaus test (Siemens Healthineers, Erlangen, Germany), and the activity of the von Willebrand factor (vWF) in citrated plasma was assessed using the partially enhanced test INNOVANCE^®^ VVF Ac (BCS XP analyzer; Siemens Healthineers, Erlangen, Germany).

### 2.5. Heart Weight Index (HWI) Calculation

After isolating the hearts, they were rinsed with 0.9% NaCl and dried with filter paper. The heart mass was then measured to calculate the HWI, which was determined as the ratio of heart mass (expressed in mg) to body mass of the rats (expressed in g) [20].

### 2.6. Preparation of the Cardiac Tissue Samples

Cardiac tissue was homogenized on ice in buffer (20 mmol/L Tris-HCl, pH 7.5, 250 mmol/L sucrose, 1% Triton X-100, with addition of protease inhibitor: 1 mmol/L phenylmethylsulfonyl fluoride (PMSF) and 1 μg/mL leupeptin). The ratio of tissue to buffer was 1:10 [9]. The homogenized sample was subjected to centrifugation at 11,200× *g* at 4 °C for 10 min to isolate cardiac tissue proteins. The collected supernatant represents the total cell lysate, in which proteins from the heart tissue were isolated. The Bradford method was employed to quantify the total protein concentration in the supernatant [21].

### 2.7. Assessing Antioxidant Enzymes in the Cardiac Tissue

Cardiac tissue enzyme activities were assessed spectrophotometrically (Shimadzu UV-2600 spectrophotometer, Kyoto, Japan) using a temperature-controlled cuvette holder. The specific activities of all tested enzymes were expressed as units of enzymatic activity per milligram of heart tissue homogenate protein (U/mg protein).

The activity of CAT in cardiac tissue lysate samples was measured according to the method of Beers and Sizer [22]. A reaction mixture containing 1.0 mL of 0.18% aqueous hydrogen peroxide (H_2_O_2_) solution, prepared by dissolving in phosphate buffer (0.05 mM, pH 7.0), was added to 2 mL of phosphate buffer (0.05 mM, pH 7.0) and 200 μL of heart tissue lysate. The change in absorbance due to the decomposition of H_2_O_2_ during the first minute of the reaction was measured at 240 nm. One unit of enzymatic activity (U) is defined as the amount of enzyme that catalyzes the degradation of 1 μmol of H_2_O_2_ to O_2_ and H_2_O per minute.

The activity of SOD in heart tissue lysate samples was determined using the adrenaline method, spectrophotometrically at 480 nm [23]. This method is based on the ability of SOD to inhibit the spontaneous autoxidation of adrenaline to adrenochrome in a basic medium (pH 10.2). The reaction mixture consisted of 1.8 mL of Tris-HCl buffer (50 mM, pH 10.2), 0.1 mL of adrenaline (0.18 g/L in 0.1 M HCl), and 0.1 mL of heart tissue homogenate. One unit of SOD activity is defined as the amount of enzyme that causes 50% inhibition of adrenaline autoxidation at 26 °C.

### 2.8. Assessing LDH and MDH Activity in the Cardiac Tissue

The activity of LDH was determined in heart tissue lysates by measuring the decrease in absorbance at 340 nm during the oxidation of NADH. The incubation mixture contained 2.90 mL of phosphate buffer in deionized water (0.1 M, pH 7.0), 0.1 mL of sodium pyruvate in deionized water (23 mM), 0.05 mL of NADH in deionized water (14 mM), and 0.01 mL of heart tissue lysate. The decrease in absorbance at 25 °C was monitored during the first 2 min of the reaction. One unit (1 U) of LDH activity catalyzes the transformation of 1 µmol of NADH per minute under the test conditions [24].

The activity of MDH was determined using the method established by Frieden and Fernandez-Sousa [25] by measuring the decrease in absorbance at 340 nm during the oxidation of NADH. The incubation mixture contained 2.90 mL of phosphate buffer in deionized water (0.1 M, pH 7.5), 0.1 mL of oxaloacetate (15 mM) in phosphate buffer (0.1 M, pH 7.5), 0.05 mL of NADH in deionized water (14 mM), and 0.01 mL of heart tissue lysate. The decrease in absorbance at 25 °C was monitored during the first 2 min of the reaction. One unit (1 U) of MDH activity catalyzes the transformation of 1 µmol of NADH per minute under the test conditions.

### 2.9. Assessing LDH and MDH Isoforms in the Cardiac Tissue

The distribution of LDH and MDH isoforms was determined using direct electrophoretic zymography, as described by Cunningham et al. [26] and Yoshimura et al. [27], respectively.

For both LDH and MDH isoform determination, native polyacrylamide gel electrophoresis was performed on a pre-prepared 7.5% native polyacrylamide gel. A volume of 5 µL of the prepared sample was applied to the gel. The sample was prepared by mixing heart tissue homogenate and a treatment buffer in a 1:1 ratio. The treatment buffer contained Tris-Cl (2.5 mL, 0.5 M, pH 6.8), glycerol (2 mL), bromophenol blue (2 mg), and distilled water to a total volume of 10 mL. After applying the samples to the gel, electrophoretic separation was carried out at 4 °C with a constant electric current of 50 mA. After approximately 90 min, when the samples reached the bottom of the gel, the electrophoresis was stopped. Upon the completion of electrophoretic separation, the gels were washed in deionized water.

For LDH isoform determination, the gels were incubated in a solution containing Li-lactate (0.2 g), NAD+ (0.015 g), phenazine methosulfate (PMS, 0.5 mg), and nitro blue tetrazolium (NBT, 0.01 g) in TRIS-Gly buffer (20 mL, 0.1 M, pH 8.3). After 30 min of incubation in the dark, isoenzymes appeared on the gel as dark blue bands. These bands result from the staining with formazan, which is produced after the reduction of NBT in the presence of the electron carrier PMS to the coenzyme NAD+. The gels were stored in 0.01 M acetic acid in the dark at 4 °C. The activity of individual isoenzymes was semiquantitatively assessed relative to the total LDH activity and expressed as a percentage (relative activity) [26].

For MDH isoform determination, the gels were incubated for 10 min in the dark at 37 °C in 20 mL of a solution containing phosphate buffer (10 mL, 0.2 M, pH 7.1) and sodium malate solution (10 mL, 0.01 M, pH 6.9), to which NAD+ (0.015 g), NBT (0.01 g), and PMS (0.5 mg) were added. After incubation in the buffer, the gels were incubated for an additional 10 min in deionized water, in the dark. Isoenzymes appeared as dark blue bands. The gels were stored in 0.01 M acetic acid in the dark at 4 °C. The activity of individual isoenzymes was semiquantitatively assessed relative to the total MDH activity and expressed as a percentage (relative activity) [27].

After zymography, the gels were scanned for densitometric analysis to determine the relative activities of LDH and MDH isoforms. The thickness and intensity of the protein bands were quantified as enzyme activities using the ImageJ software package (ImageJ 1.48v, National Institutes of Health, Bethesda, MD, USA). Densitometric data were normalized to 1 µg of total heart tissue protein.

### 2.10. Histomorphometric and Immunohistochemical Analysis of the Cardiac Tissue

The heart tissue underwent meticulous preparation, starting with orientation and fixation in 4% neutral-buffered formaldehyde for a minimum of 24 h. Following fixation, the tissue was dehydrated, embedded in paraffin, and sectioned into 5 μm slices using a microtome (Leica Reinhart Austria and Leica SM2000 R, Heidelberg, Germany) until the full thickness of the heart wall was visible. These sections were subsequently stained with hematoxylin and eosin, enabling the examination of cardiac morphology. For morphological assessment, measurements were taken from the endocardium to the epicardium, measuring the thickness of the left ventricle (LV) wall, right ventricle (RV) wall, and interventricular septum (IVS), with ten measurements recorded per heart.

Additionally, tissue sections (4 μm thick) obtained from the heart molds underwent immunohistochemical staining to detect proliferation markers: marker of proliferation Kiel 67 (Ki-67) and proliferating cell nuclear antigen (PCNA). Antigen retrieval was performed using heat-induced epitope retrieval (HIER), utilizing either citrate buffer (pH 6) in a microwave at 21 °C at maximum power (800 W) or Tris-EDTA buffer (pH 9). Antibodies were applied at an appropriate dilution of 1:200 for both Ki-67 Clone SP6 (Thermo Scientific Lab Vision RM-9106-S0, Thermo Fisher Scientific, Waltham, MA, USA) and PCNA Clone PC10 (Thermo Scientific Lab Vision MS-106-P0). Immunohistochemical reactions were developed using secondary antibodies from the Thermo Scientific Lab Vision Quanto HRP DAB TL-125-QHD kit, followed by visualization with the chromogen diaminobenzidine (DAB) and counterstaining with Mayer’s hematoxylin (Biognost, HEMML-OT-1L, Zagreb, Croatia). Analysis of immunoreactivity for Ki-67 and PCNA was conducted to assess the proliferative activity of cardiomyocytes across all experimental groups, with the results expressed as a percentage of immunoreactive cells out of 100 counted cardiomyocytes.

The slides were examined using a microscope (Olympus BX 41, Tokyo, Japan) and captured with a wide zoom digital camera (Olympus C-5060-ADU, Tokyo, Japan) coupled with the Leica LAS software package (version 4.4.0). Subsequent morphometric analysis of the photomicrographs was carried out using the ImageJ software package (ImageJ 1.48v, National Institutes of Health, Bethesda, MD, USA), with all measurements reported in micrometers (μm) or as percentages.

### 2.11. Statistical Analysis

Statistical analyses were conducted using one-way ANOVA with Tukey’s post hoc test for parametric data, and if there was no normality of the data distribution, the Kruskal–Wallis test followed by the Mann–Whitney U test was used. Data are presented as mean ± SEM or median (range from minimum to maximum). The analyses were performed using the SPSS 19.0 software package for Windows. A *p*-value of less than 0.05 was considered statistically significant.

## 3. Results

All experimental animals survived over the four-week study period. When presenting the results, for clarity, we will display all groups. However, the impact of diabetes mellitus will not be significantly analyzed, considering that its influence on tested parameters has been addressed in our previous work [9].

### 3.1. Body Mass and Heart Weight Index

**Figure 1:**
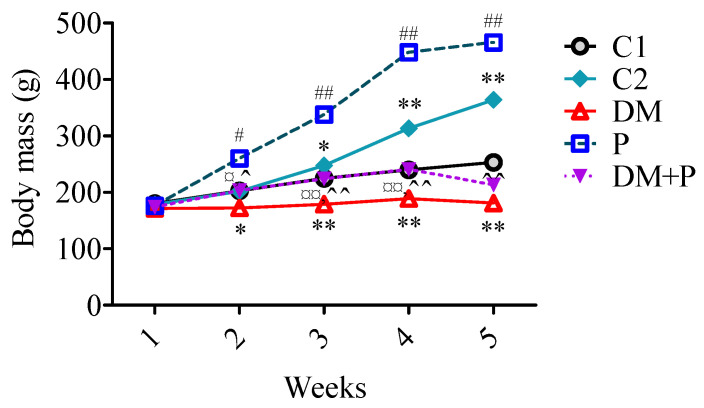
Effect of pyridoxine treatment on body mass over 4 weeks. Measurements were taken at five time points: (1) the start of the experimental period, (2) the first week, (3) the second week, (4) the third week, and (5) the fourth week. * *p* < 0.05 versus (vs.) C1, # *p* < 0.05 vs. C2, ¤ *p* < 0.05 vs. DM, ^ *p* < 0.05 vs. P, ** *p* < 0.01 vs. C1, ## *p* < 0.01 vs. C2, ¤¤ *p* < 0.01 vs. DM, ^^ *p* < 0.01 vs. P.

**Figure 2:**
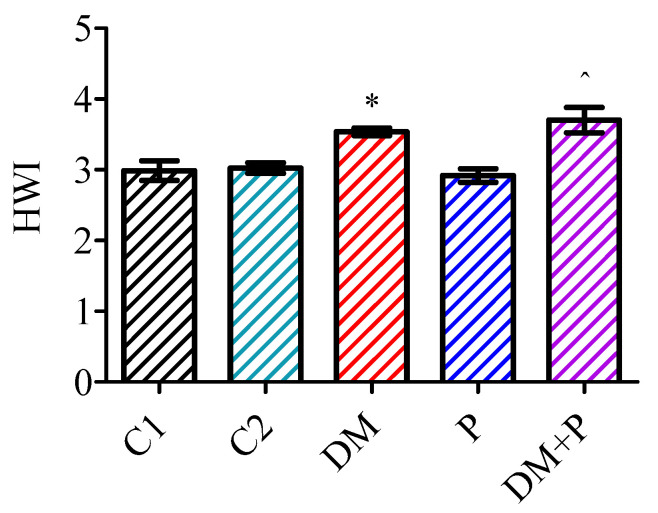
Heart weight index (HWI). * *p* < 0.05 vs. C1, ^ *p* < 0.05 vs. P.

Pyridoxine treatment significantly increased the body mass in both diabetic and healthy rats over four weeks. Healthy rats treated with pyridoxine showed a 30% increase in body mass (*p* < 0.001). However, no significant differences were observed between diabetic rats treated with pyridoxine and those untreated at the end of the experimental period (*p* = 0.564, Figure 1). Pyridoxine did not affect the heart weight index (HWI) in either diabetic or healthy rats (Figure 2).

### 3.2. Biochemical Parameters

**Figure 3:**
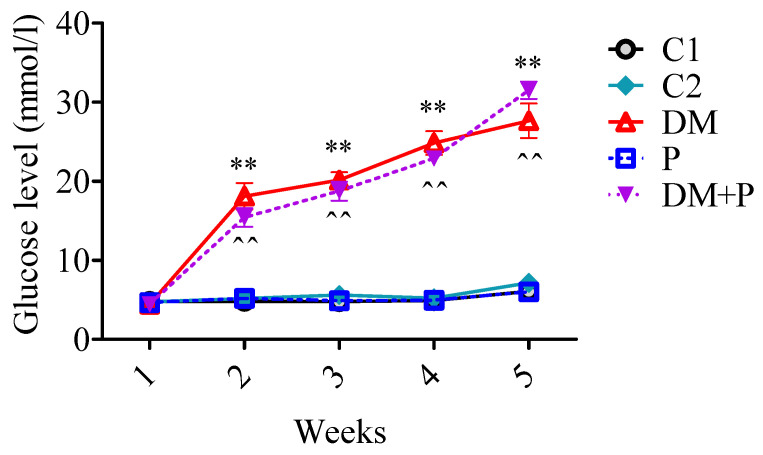
Glucose level over 4 weeks. Measurements were taken at five time points: (1) the first week, (2) 72 h after streptozotocin administration, (3) the second week, (4) the third week, and (5) the fourth week. ** *p* < 0.01 vs. C1, ^^ *p* < 0.01 vs. P.

The glucose level did not differ between the tested groups before the streptozotocin treatment (*p* = 0.066); however, an increased glucose level was found in all animals that received streptozotocin 72 h after the treatment (DM = 17.2 ± 0.65 mmol/L and DM + P = 15.4 ± 1.16 mmol/L). The glucose level in both diabetic groups remained elevated without differences until the end of the experimental period (Figure 3).

The administration of pyridoxine did not affect the levels of homocysteine, LDH, fibrinogen, and vWF in healthy rats. In diabetic rats, pyridoxine significantly reduced homocysteine levels by about 36% compared to untreated diabetic rats (Table 1).

Comparing lipid profile parameters, pyridoxine significantly increased TC and LDL-C levels in healthy rats, while in diabetic rats, it reduced TC, HDL-C, and TG levels but increased LDL-C levels (Table 2).

### 3.3. Activities of Antioxidant Enzymes in the Cardiac Tissue

**Figure 4:**
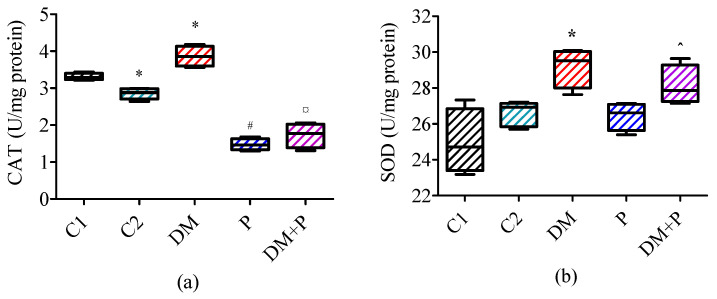
Activities of antioxidant enzymes in the cardiac tissue: (**a**) catalase (CAT) activity and (**b**) superoxide dismutase (SOD). * *p* < 0.05 vs. C1, # *p* < 0.05 vs. C2, ¤ *p* < 0.05 vs. DM, ^ *p* < 0.05 vs. P.

CAT and SOD activity in cardiac tissue homogenate differed significantly among the groups (*p* = 0.001 and *p* = 0.007, respectively). The administration of pyridoxine was followed by a decrease in CAT activity by about 50% in the healthy rats (*p* = 0.014), as well as in the rats with induced DM (*p* = 0.021, Figure 4a). SOD activity was not significantly affected by pyridoxine treatment (*p* = 0.624 for P vs. C2, *p* = 0.149 for DM + P vs. DM, Figure 4b).

### 3.4. Activities of LDH and MDH in the Cardiac Tissue

**Figure 5:**
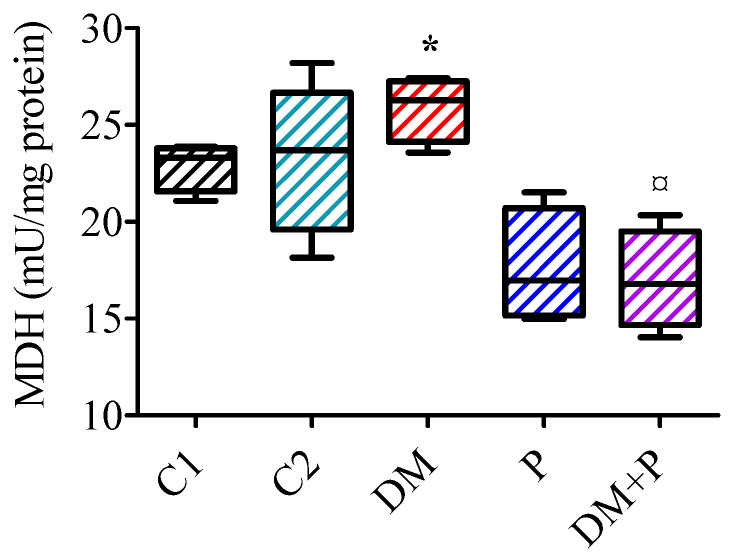
Activity of malate dehydrogenase (MDH) in the cardiac tissue. * *p* < 0.05 vs. C1, ¤ *p* < 0.05 vs. DM.

LDH activity did not vary among the groups (*p* = 0.259; C1 group: 15.31 (13.60–16.28) μU/mg protein, C2: 13.65 (12.17–14.10) μU/mg protein, DM: 16.92 (12.30–18.41) μU/mg protein, P: 15.66 (13.71–16.14) μU/mg protein, DM + P: 15.74 (12.20–16.16)). However, MDH activity varied significantly (*p* = 0.010), with pyridoxine treatment decreasing MDH activity by about 37% in the DM + P group compared to the DM group (*p* = 0.021, Figure 5).

### 3.5. Activities of LDH and MDH Isoforms in the Cardiac Tissue

**Figure 6:**
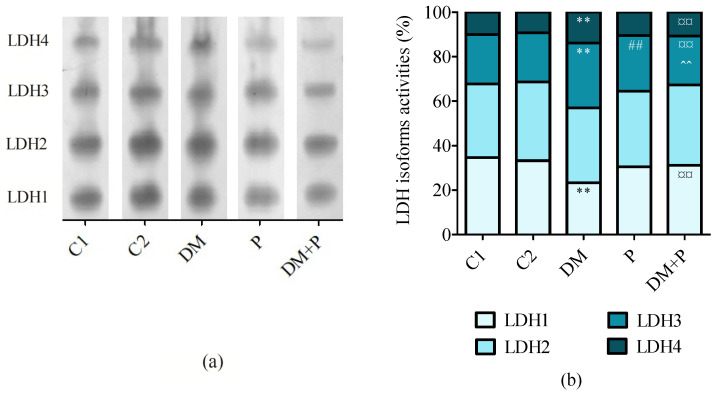
(**a**) Representative zymograms of lactate dehydrogenase isoform activities (LDH1, LDH2, LDH3, and LDH4) detected in rat heart tissue by native polyacrylamide gel electrophoresis and (**b**) relative activity of LDH isoform in the cardiac tissue. ** *p* < 0.01 vs. C1, ## *p* < 0.01 vs. C2, ¤¤ *p* < 0.01 vs. DM, ^^ *p* < 0.01 vs. P.

**Figure 7:**
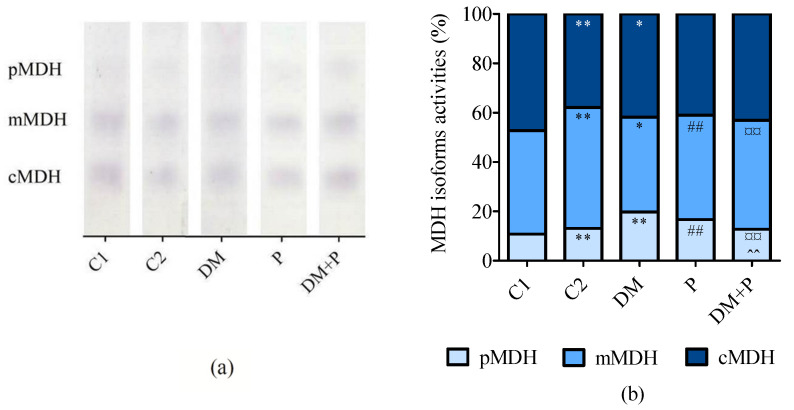
(**a**) Representative zymograms of malate dehydrogenase (MDH) isoform activities detected in rat heart tissue by native polyacrylamide gel electrophoresis and (**b**) relative activity of MDH isoform in the cardiac tissue. pMDH—peroxisomal MDH; mMDH—mitochondrial MDH; cMDH—cytosolic MDH. * *p* < 0.05 vs. C1, ** *p* < 0.01 vs. C1, ## *p* < 0.01 vs. C2, ¤¤ *p* < 0.01 vs. DM, ^^ *p* < 0.01 vs. P.

All samples of heart tissue exhibited four LDH isoforms (LDH1-4, Figure 6a) and three MDH isoforms (pMDH, mMDH, cMDH, Figure 7a). There was no statistically significant difference in LDH2 relative activity. Statistically significant differences in LDH1, LDH3, and LDH4 isoform activities were noticed across the groups (*p* < 0.001 for all isoforms, Figure 6b). Pyridoxine mitigated diabetes-induced alterations in LDH isoform activities, increasing LDH1 (*p* < 0.001) and reducing LDH3 and LDH4 activities in diabetic rats (*p* < 0.001 for both, Figure 6).

MDH isoform activities also showed significant variation (*p* < 0.001 for all isoforms, Figure 7b). Pyridoxine increased pMDH and decreased mMDH activity in healthy rats compared to untreated controls (*p* < 0.001 for both). In diabetic rats, pyridoxine decreased pMDH and increased mMDH activity in comparison to untreated diabetic rats (*p* < 0.001 and *p* = 0.001, respectively).

### 3.6. Histomorphometric and Immunohistochemical Analysis

**Figure 8:**
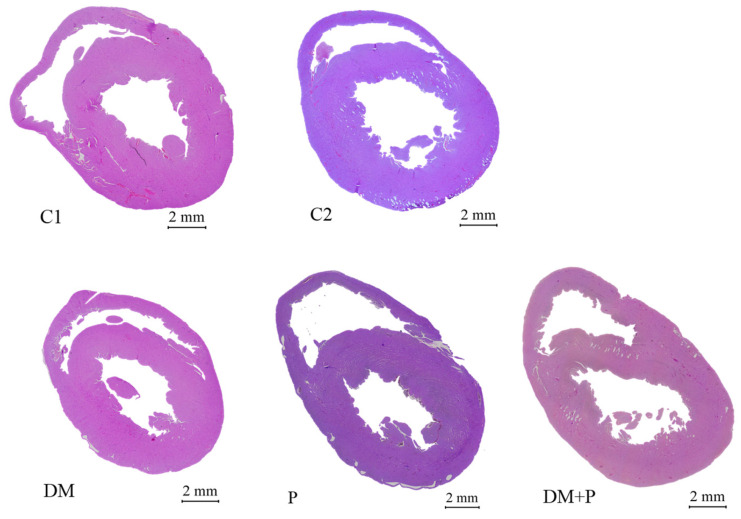
Histological sections of rats’ hearts in tested groups (hematoxylin–eosin staining at 50× magnification).

Cardiomyocytes and myocardial fibers exhibited physiological morphology across all groups (Figure 8). In healthy rats, pyridoxine increased LV wall thickness and IVS thickness, but this effect was not observed in diabetic rats (Table 3).

**Figure 9:**
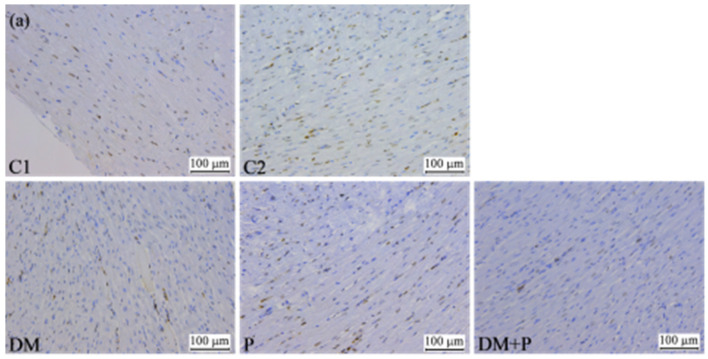

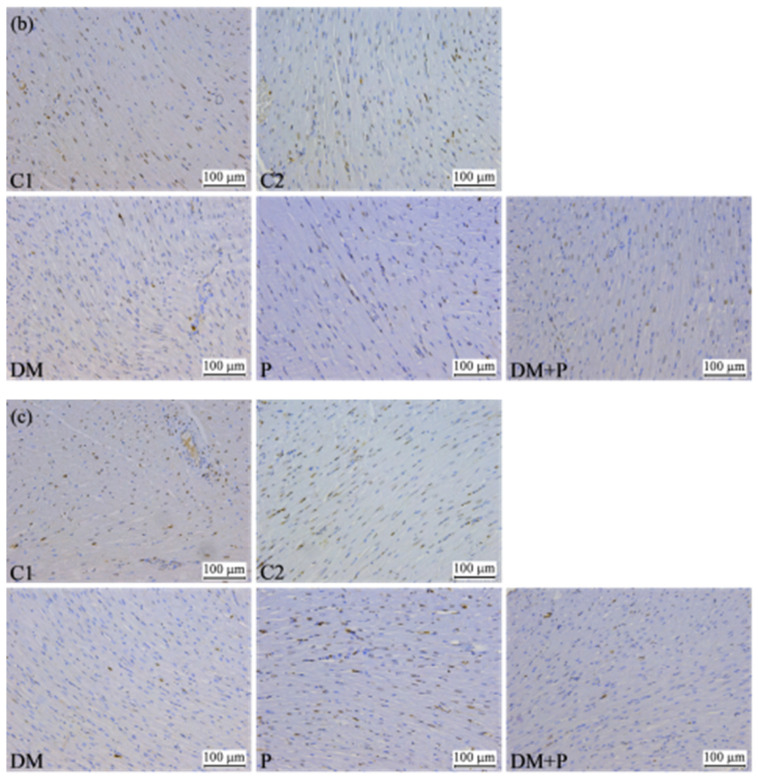
Effect of diabetes mellitus and pyridoxine administration on immunohistochemical expression of PCNA in (**a**) left ventricle, (**b**) right ventricle, and (**c**) interventricular septum of rat heart. PCNA-positive nuclei of cardiomyocytes stained brown and PCNA-negative nuclei of cardiomyocytes stained blue can be observed (magnification 200×).

The positivity of Ki-67 was observed in certain cells of the cardiac tissue without a statistically significant difference between the tested groups. The positivity of PCNA was significantly lower in the LV wall, RV wall, and IVS in the group of rats with DM compared to the control (C1) group. The administration of pyridoxine reduced PCNA positivity in the LV (*p* = 0.021) and IVS (*p* = 0.021) but increased it in the RV (*p* = 0.026) in healthy rats. In rats with DM, pyridoxine treatment led to an increase in PCNA positivity in the RV (*p* = 0.021) without affecting LV (*p* = 0.243) and IVS (*p* = 0.278) PCNA positivity (Table 4, Figure 9).

## 4. Discussion

DM over 28 days leads to various organ injuries and increases oxidative stress, which is considered a crucial mechanism in its pathogenesis, and can cause endothelial cell dysfunction and the progression of atherosclerosis, highlighting the importance of reducing oxidative stress in its management [5,6,7,8,9,28]. In our previous research, we found that DM induces numerous cardiometabolic changes in rats within just four weeks [9,10]. This study aimed to evaluate the effects of pyridoxine supplementation in both healthy and diabetic rats, since we have demonstrated that folic acid supplementation during a four-week period exhibits potent antidiabetic, antioxidant, and many other positive effects [9,10].

In this research, the glucose level was measured 72 h after the injection of streptozotocin, and all the experimental animals had hyperglycemia. Our previous research demonstrated that the administration of 100 mg/kg streptozotocin induces DM type I, since it increased glucose levels and decreased insulin levels. Also, it increased the Homeostasis Model Assessment of Insulin Resistance (HOMA-IR) [9]. In that study, strong antidiabetic effects of folic acid were obtained [9]; however, in the present study, pyridoxine did not influence glucose levels. One of the characteristics of DM type I is a decrease in body mass [29], which was obtained in both diabetic groups, without any statistical difference between them. The administration of pyridoxine resulted in increased body weight in healthy rats, while it did not affect body weight in rats with DM. Similarly, Kalicki et al. [30] demonstrated that vitamin B6 supplementation led to a significant increase in body weight in rats. It was shown that vitamin B6 increased muscle mass and improved the synthesis of muscle proteins [31], suggesting that the increased intake of this vitamin may also lead to weight gain.

Oxidative stress and inflammation associated with diabetes contribute to the hypertrophy of the heart and cardiomyocytes, which are indicators of diabetic cardiomyopathy [32,33]. In our study, cardiac hypertrophy in diabetic rats was validated by observing elevated heart weight index (HWI) values. The higher HWI in the diabetic group suggested that the reduction in heart mass did not correlate with a significant loss of body mass. Similar findings regarding cardiac hypertrophy, assessed through HWI, have been reported by other researchers [20,34]. The administration of pyridoxine did not affect the value of the HWI in either healthy or DM rats. A higher value of the HWI was observed in the DM + P group compared to the P group.

It is widely recognized that an elevated serum LDH concentration is a sign of cardiomyocyte injury [35], but even though cardiac hypertrophy was demonstrated, serum LDH levels were not increased. The LDH level in serum did not vary between the tested groups, demonstrating that cardiomyocyte membrane damage did not occur. Correspondingly, there were no necrosis signs confirmed by histological analysis. This absence of cardiomyocyte membrane damage suggests that despite cardiac hypertrophy, significant tissue injury may not have occurred. However, in some experiments that lasted 8 weeks, increased serum LDH was obtained [20,34], indicating that our results may be due to the shorter experimental period. Since apoptosis, or programmed cell death, plays a vital role in cardiac tissue homeostasis and pathological conditions, assessing the markers of apoptosis could provide further insights into the cellular response to diabetic conditions. Integrating such analyses in further research could enhance our understanding of diabetic cardiomyopathy progression and potential therapeutic interventions.

Our previous study showed that when comparing lipid profile parameters, a statistically significant increase was observed in the DM group [9]. Unexpectedly, the pyridoxine treatment significantly reduced HDL-C and increased LDL-C, TC, and TG levels in the diabetic rats. In one study, it was shown that pyridoxine administered via oral gavage at a dose of 20 mg/kg increased HDL-C and decreased in TC and TG; however, when pyridoxine was administered at a lower dose (10 mg/kg), it had no effects on lipid profile parameters [36]. Also, reduced HDL-C and increased LDL-C and TC levels were observed in the second control group (C2) compared to the first control group (C1). The C2 group was exposed to daily stress due to physical restraint used during i.p. substance administration compared to the C1 group. It has been shown that animal restraint leads to an increase in stress parameters and affects the lipid profile by lowering HDL-C and increasing LDL-C levels [37,38], as shown in our study. Similarly, daily physical restraint during i.p. pyridoxine administration may be a factor in the changes in lipid profile parameters in the DM + P group.

Upon measuring biochemical serum parameters, it was observed that the serum homocysteine level experienced a significant decrease in the DM + P group, suggesting a potential antioxidative effect of pyridoxine, as elevated serum homocysteine levels typically indicate increased oxidative stress [39]. High homocysteine levels are commonly associated with nutritional deficiencies in essential vitamin cofactors such as vitamin B6, folate, and vitamin B12, all crucial for the metabolism of homocysteine [40]. These vitamins are crucial for lowering total homocysteine levels and improving endothelial function [41]. Our results showed that pyridoxine administration did not influence homocysteine levels in healthy rats. Lindschinger et al. [42] conducted a pilot study on a healthy population, demonstrating that the use of a natural vitamin B complex, which also contained pyridoxine, resulted in a notable decrease in serum homocysteine levels. However, similar to our results, this effect was not observed after the application of a synthetic vitamin B complex. Our previous publication demonstrated decreased fibrinogen in diabetic rats [9]. Fibrinogen is a liver product and hemostatic factor. Since our previous results may indicate impaired liver function, in our second study, we examined liver function enzyme activities in rats with DM. We observed that all tested enzymes, alkaline phosphatase, aspartate aminotransferase, and alanine aminotransferase, were significantly higher in DM rats [10]. Similarly, Chayarop et al. [43] showed significantly higher serum alkaline phosphatase in DM rats. All these results indicate possible liver damage in a diabetic state. After pyridoxine application, the fibrinogen level was significantly increased in diabetic rats, pointing to the protective effects of pyridoxine against liver damage linked to hyperglycemia. Pyridoxine treatment did not influence previously increased vWF in diabetic rats, as well as in healthy rats.

Another hemostatic parameter, the von Willebrand factor (vWF), is a critical endothelial marker involved in hemostasis under physiological conditions. Primarily secreted by endothelial cells, the vWF can serve as a biomarker for endothelial injury and dysfunction in pathological states such as vasculitis [44,45]. Studies have demonstrated elevated vWF levels in patients with DM type 2 or insulin resistance [44,45,46], linking these increased levels to a higher risk of cardiovascular diseases [44].

Recent research has highlighted the significant role of increased oxidative stress and altered antioxidant capability in the pathogenesis of DM [5], as well as in the progression of neuronal, vascular, renal, and ophthalmological complications associated with diabetes [6]. Prolonged hyperglycemia leads to the generation of reducing sugars that react with proteins and lipids, thereby increasing the generation of reactive oxygen species (ROS). This process also induces the production of advanced glycation end products, which serve as markers of oxidative stress in plasma due to hyperglycemia [6,47]. Our previous research already demonstrated that the cardiac tissue antioxidant enzyme activities were increased in diabetic rats [9]. Similarly, this was demonstrated by other authors [28,48]. These enzymes play a protective role against damage induced by oxygen free radicals; thus, their increased activity can be interpreted as an adaptive response to elevated oxidative stress [49]. Pyridoxine treatment had a positive effect on the healthy and diabetic rats, since it decreased the CAT activity. However, it had no influence on SOD activity. SOD catalyzes the conversion of superoxide anion to H_2_O_2_. The obtained H_2_O_2_ is the substrate of CAT [49]. The administration of pyridoxine led to a reduction in CAT activity. This can be connected with the fact that pyridoxine scavenges H_2_O_2_ and removes the CAT substrate. Vitamin B6 has been shown to inhibit the formation of superoxide radicals, act as a singlet oxygen quencher, and directly interact with peroxide radicals to inhibit lipid peroxidation. Additionally, it influences the conversion of homocysteine to cysteine, a crucial step in the glutathione-dependent antioxidant defense system [14,50,51,52]. However, the chemical mechanism of its antioxidant action is not completely known [50]. In general, ROS can be scavenged by hydroxyl and amine groups that are electron-donating groups [53]. Vitamin B6 consists of a pyridine ring with the substitution of both the hydroxyl and amine groups. Therefore, it is possible that the antioxidant potential of vitamin B6 results from the presence of these two groups in its structure [52]. Regardless, positive antioxidant effects of pyridoxine are demonstrated in the cardiac tissue. In addition to the positive effect of pyridoxine on antioxidant enzymes, we also showed that the application of folic acid had a useful effect on rats with DM. It reduced the activities of both antioxidant enzymes, CAT and SOD. Interestingly, this effect was not observed in healthy rats [9]. Comparable results were noted in another investigation, where the combined administration of vitamin B6 and folic acid did not influence SOD activity in healthy rats [54].

LDH and MDH are members of 2-ketoacid: the NAD(P)-dependent dehydrogenases group. LDH catalyzes a final step in glycolysis, while MDH plays a role in the tricarboxylic acid cycle [55]. Given their central roles in metabolism, the assessment of their activities in cardiac tissue can offer valuable insights into the metabolic profile. Although LDH activity did not differ across the groups, a comparison of LDH isoforms revealed that pyridoxine treatment in diabetic rats increased LDH1 activity and reduced LDH4 activity compared to the values observed in the control group. LDH catalyzes the oxidation of lactate to pyruvate in aerobic conditions, while the reduction of pyruvate to lactate is also catalyzed by LDH, and this reaction occurs in oxygen deficiency states. There are five isoforms of LDH. Based on the cellular environment and oxygen supply, the production of isoenzymes is different. In aerobic conditions, more LDH1 and LDH2 are synthesized, while in hypoxic conditions, there is a tendency for LDH4 and LDH5 production [14,15]. The decreased activity of LDH1 and increased activity of LDH4 suggest decreased pyruvate formation due to inadequate oxygen supply and hypoxic conditions [15]. According to these facts, our results might point out that there was insufficient oxygen supply in the cardiac tissue of diabetic rats that led to a shift towards anaerobic metabolism [9] and that pyridoxine administration returned the metabolism to aerobic.

LDH is involved in glycolysis, directly by pyruvate reduction and indirectly by nicotinamide adenine dinucleotide (NAD+) synthesis. The control point of glycolysis is the production of 1,3—bisphosphoglycerate after the oxidation of glyceraldehyde 3-phosphate. This reaction requires the availability of the cofactor NAD+. Cells can provide NAD+ via LDH activity that converts pyruvate to lactate with the simultaneous regeneration of NAD+. The cytosolic isoform of MDH is another enzyme that can increase NAD+ production in hypoxic conditions. MDH catalyzes the reduction of oxaloacetate to malate in the presence of NADH [56]. This reaction’s products, NAD+ and malate, are involved in glycolysis regulation. The obtained NAD+ is used as a cofactor for the progression of glycolysis [57], while malate transforms into pyruvate by malic enzyme activity [58], and the generated pyruvate becomes an LDH substrate and participates in NAD+ synthesis and consequently promotes glycolysis. As a result, MDH directly through the production of NAD+ and indirectly through the production of malate supports glycolysis [56]. Thus, the MDH activity can be used as a parameter for the estimation of metabolic changes in the mitochondria and cytoplasm that can be expected in a metabolic disorder such as DM. Pyridoxine treatment significantly decreased the total MDH activity that was increased in diabetic rats. DM is characterized by gluconeogenesis in which a rise in the activity of MDH is anticipated [59,60]. Except our research, there is no research about the effect of pyridoxine on the activity of MDH in the cardiac tissue. An increased activity of pMDH and decreased mMDH and cMDH isoform activities were observed in rats with DM [9]. The positive effects of pyridoxine administration in rats with DM are reflected by a reduction in pMDH activity and a rise in mMDH activity. Other studies have also confirmed decreased mMDH activity in rats with DM, which demonstrates that there is mitochondrial damage [61,62]. It was demonstrated that ROS are implicated in causing damage to the mitochondria and in the decrease in the activities of citric acid cycle enzymes [61]. Other researchers have shown alterations in various peroxisomal enzyme activities in diabetic conditions, although they did not specifically investigate MDH activity [63]. As already mentioned, in addition to the increased activity of pMDH, the activity of CAT, which is a peroxisomal enzyme, was also increased in rats with DM [9]. In diabetic conditions, peroxisomal activation may be the consequence of the increased supply of fatty acids as well as heightened oxidative stress [63,64]; these findings align with our study results.

Although there were many biochemical changes in the conditions of DM, histologically, the cardiomyocytes had a physiological morphology and they and the myocardial fibers were arranged regularly in all tested groups. IVS and LV thicknesses were increased in the P group; however, there was no influence on these parameters in rats with DM.

The frequently used method for determining cell proliferative activity is the determination of Ki-67 and PCNA positivity by the application of immunohistochemical techniques [65]. In this study, to ascertain whether changes in the proliferative activity of cardiomyocytes had occurred, the immunohistochemical staining of preparations for Ki-67 and PCNA was performed. Positivity for Ki-67 was observed only in certain cells of the cardiac tissue. DM, as well as the administration of pyridoxine to healthy rats and rats with DM, did not affect Ki-67 activity. Decreased positivity of PCNA was observed in rats with DM in the LV wall, RV wall, and IVS. The positive effects of pyridoxine administration were reflected in the increase in PCNA positivity in the RV in both healthy rats and rats with induced DM. However, in healthy rats, a decrease in PCNA positivity was also observed in the LV and IVS. Ki-67 is expressed in all cell cycle active phases, except in the G0 phase; thus, its expression indicates that Ki-67 may be a proliferation marker, but its expression may not always indicate cell duplication [65,66]. PCNA acts as a cofactor for deoxyribonucleic acid polymerase delta, playing a crucial role in the proliferation of cells [65]. It is a nuclear non-histone protein necessary for the synthesis of DNA and a DNA polymerase alpha accessory protein. It is increased during the G1/S phase [66]. Surprisingly, our study obtained a higher percentage of PCNA-positive cardiomyocytes compared to the percentage of Ki-67-positive cardiomyocytes. Also, other studies conducted on tumor tissues have shown no correlation between Ki-67- and PCNA-positive cells, and the percentage of PCNA-positive cells was significantly higher than the percentage of Ki-67-positive cells [66,67]. PCNA is crucial in nucleic acid metabolism, as it participates in both DNA replication and reparation. It is believed that growth factors or DNA damage may promote a higher PCNA level, which does not always correlate directly with cell proliferation [66]. This could explain why there might be a higher percentage of PCNA-positive cells compared to Ki-67-positive cells.

## 5. Conclusions

Our previous studies revealed significant cardiometabolic changes in rats within a short timeframe after the onset of DM. Following the antidiabetic and other promising results of folic acid supplementation in our earlier research, our current study explored the effects of pyridoxine supplementation in both healthy and diabetic rats. Our findings highlight the positive effects of pyridoxine administration on the complex interplay between oxidative stress, antioxidant enzymes, and metabolic changes in diabetic cardiomyopathy. However, further investigations are warranted to elucidate the underlying mechanisms and translational implications of these findings.

## Figures and Tables

**Table 1:** Cardiovascular and hemostatic biomarkers in serum or plasma of experimental animals.

Parameters	Groups (Mean ± SEM)					*p* Value
	C1	C2	DM	P	DM + P	
Homocysteine (mmol/L)	9.0 ± 0.46	10.4 ± 0.64	8.3 ± 0.39	11.1 ± 0.36	5.3 ± 0.29 ¤¤,^^	<0.001
LDH (U/L)	3887.1 ± 358.5	4438.4 ± 331.7	3913.6 ± 249.3	3291.9 ± 169.3	4977.1 ± 449.1 ^^	0.014
Fibrinogen (g/L)	2.6 ± 0.3	2.2 ± 0.1	1.2 ± 0.2 *	1.7 ± 0.1	1.9 ± 0.3	0.001
vWF (% d.N.)	214.3 ± 30.4	99.4 ± 4.3	467.9 ± 43.6 **	196.7 ± 11.4	376.6 ± 38.5 ^^	<0.001

Note: C1 and C2, control groups; DM, diabetes mellitus; P, pyridoxine; LDH, lactate dehydrogenase; vWF, von Willebrand factor; % d.N., percentage of normal value; * *p* < 0.05 vs. C1, ** *p* < 0.01 vs. C1, ¤¤ *p* < 0.01 vs. DM, ^^ *p* < 0.01 vs. P.

**Table 2:** Lipid profile parameters in serum of experimental animals.

Parameters	Groups (Mean ± SEM)					*p* Value
	C1	C2	DM	P	DM + P	
TC (mmol/L)	1.38 ± 0.06	1.53 ± 0.07	2.32 ± 0.13 **	1.78 ± 0.06 #	1.98 ± 0.09 ¤	<0.001
HDL-C (mmol/L)	1.12 ± 0.05	0.64 ± 0.03 **	1.51 ± 0.11 **	0.57 ± 0.03	0.73 ± 0.04 ¤¤,^^	<0.001
LDL-C (mmol/L)	0.09 ± 0.01	0.53 ± 0.06 **	0.28 ± 0.06 **	0.77 ± 0.07 #	0.52 ± 0.11 ¤,^	<0.001
TG (mmol/L)	0.58 ± 0.04	0.78 ± 0.04 *	2.03 ± 0.22 **	0.96 ± 0.10	1.77 ± 0.29 ¤	<0.001

Note: C1 and C2, control groups; DM, diabetes mellitus; P, pyridoxine; TC, total cholesterol; HDL-C, high-density lipoprotein; LDL-C, low-density lipoprotein; TG, triglycerides; * *p* < 0.05 vs. C1, # *p* < 0.05 vs. C2, ¤ *p* < 0.05 vs. DM, ^ *p* < 0.05 vs. P, ** *p* < 0.01 vs. C1, ¤¤ *p* < 0.01 vs. DM, ^^ *p* < 0.01 vs. P.

**Table 3:** Histomorphometric parameters of rat heart at middle cross-section level.

Parameters	Groups (Mean ± SEM)					*p* Value
	C1	C2	DM	P	DM + P	
LV wall thickness (μm)	2528.7 (2137.8–3264.7)	2242.6 (1810.0–2290.6)	2117.6 (2033.3–2409.6)	2515.8 (2440.0–3262.7) #	2055.6 (1654.0–2432.6) ^	0.039
IVS thickness (μm)	2429.5 (2113.5–2782.5)	2014.5 (1709.5–2394.5)	1875.8 (1666.6–2047.9) *	2635.4 (2594.2–3330.7) #	1781.4 (1703.0–1987.2) ^	0.007
RV wall thickness (μm)	878.9 (648.3–994.7)	821.8 (712.9–963.4)	765.3 (478.1–848.9)	1049.3 (877.1–1208.7)	779.7 (561.7–1219.0)	0.192

Note: C1 and C2, control groups; DM, diabetes mellitus; P, pyridoxine; LV, left ventricle; IVS, interventricular septum; RV, right ventricle; * *p* < 0.05 vs. C1, # *p* < 0.05 vs. C2, ^ *p* < 0.05 vs. P.

**Table 4:** Immunohistochemical parameters of rat heart.

Parameters	Groups [Median (Minimal–Maximal Value)]					
	C1	C2	DM	P	DM + P	*p* Value
PCNA + LV (%)	4 (3–7)	5.5 (3–7)	2 (1–3) *	4 (2–8) #	3.5 (2–5)	0.008
PCNA + RV (%)	6 (5–7)	4.5 (4–6)	2.5 (1–4) *	5 (4–8) #	3.5 (3–6) ¤	0.007
PCNA + IVS (%)	4 (4–5)	6.5 (3–8) *	1.5 (0–3) *	4 (3–5) #	2 (1–3) ^	0.003

Note: C1 and C2, control groups; DM, diabetes mellitus; P, pyridoxine; LV, left ventricle; IVS, interventricular septum; RV, right ventricle; * *p* < 0.05 vs. C1, # *p* < 0.05 vs. C2, ¤ *p* < 0.05 vs. DM, ^ *p* < 0.05 vs. P.

## Data Availability

The data used to support the findings of this study are available from the corresponding author upon request.
